# Children’s Interpretations of Numerically Quantified Expression Ambiguities: Evidence from Quantified Noun Phrases and Bare Cardinals †

**DOI:** 10.3390/children11070756

**Published:** 2024-06-21

**Authors:** Marilena Mousoulidou, Kevin B. Paterson

**Affiliations:** 1 Department of Psychology, Neapolis University Pafos, 8042 Paphos, Cyprus; marilena.mousoulidou@nup.ac.cy; 2 Department of Neuroscience, Psychology and Behaviour, The University of Leicester, Leicester LE1 7RH, UK

**Keywords:** children’s language comprehension, numerically quantified expressions, ambiguities, integration

## Abstract

Understanding how children comprehend text by forming links between sentences has been the focus of research for decades. Such research has consistently shown that children use anaphors and resolve ambiguities in a different manner than adults. The present study examined a less-studied anaphoric reference that arises when two numerically quantified expressions (e.g., “three cats… two cats…”) are used in the text. Focusing on 249 six- to eight-year-old children and 50 adults for comparison, the study employed a picture selection task across six experiments to assess interpretative preferences in ambiguous and unambiguous discourses containing numerically quantified expressions. The findings indicate a pronounced difference in interpretative strategies: unlike adults, who predominantly adopted an anaphoric subset reading, children showed a consistent preference for the non-anaphoric reading, even in contexts explicitly disambiguated towards this interpretation. This preference persisted across various experimental manipulations, highlighting challenges in text integration and comprehension among children. Contributing to the developmental trajectory of language comprehension, this study underscores the complexity of cognitive development and linguistic interpretation, revealing significant developmental differences in processing numerically quantified expressions and anaphoric references within discourse.

## 1. Introduction

For decades, a pivotal area of scholarly interest has been how children successfully comprehend text. Much of this research has focused on identifying the skills and cognitive processes involved and how they progress developmentally. Such research has highlighted the complexity of reading comprehension [1,2,3,4,5,6,7,8]. Reading comprehension is a complex construct that largely depends on readers’ ability to establish connections between the information they are currently processing and the preceding discourse context. Explicit and implicit references play a crucial role in this process, as they establish coherence and facilitate understanding of the relationships between entities or elements in the text [9,10]. Much of the existing literature comparing adults with children highlights differences in the use of implicit and explicit references in these two groups [11,12,13,14,15,16]. Such research primarily focuses on how children produce and interpret anaphors (i.e., the definite article, pronouns, and inferences) and resolve referential ambiguities. To enhance our understanding of children’s comprehension strategies, the present study focuses on how six- to eight-year-olds interpret ambiguities that arise when two numerically quantified expressions are used within a discourse.

In terms of children’s use of anaphoric devices to connect different parts of the text, findings suggest that young children, unlike adults, use pronouns, definite references, and inferences in a non-adult manner, with these differences decreasing with age. Developmental studies examining pronouns show differences in how children use and produce them compared to adults. Children may allow a non-anaphoric interpretation of pronouns and interpret them without linking them to previously established referents in the discourse context [13,16], use pronouns ambiguously in their narratives and use non-linguistic cues like pointing to disambiguate their referents [17,18], inconsistently use pronouns to link different parts of a text [11,15], and are less successful than adults in using the grammatical constraints and discourse structure to find the correct antecedent for a pronoun among multiple possible options [12,14,19]. Similarly, developmental research that examined children’s production and interpretation of the definite article shows that children often incorrectly use the definite article to refer to one out of many identical objects or items or use the definite article to refer to an object or item that has not yet been established in the context [13,20,21,22,23,24]. Lastly, numerous studies show that, while adults automatically draw inferences to connect information in different sentences [25], young children and children who are poor comprehenders are less successful in inferring information from text, with this ability developing with age [2,3,4,7,26,27,28,29,30].

Children’s challenges with anaphora resolution and connecting different sentences become especially apparent when children face ambiguous linguistic contexts. Ambiguities occur when there are multiple possible interpretations of a word, sentence, phrase, or discourse [31]. To grasp the intended meaning, readers must clarify these ambiguities to maintain a coherent, unified mental representation of the text. The inability to resolve such ambiguities may result in comprehension failure or misunderstanding of the text. Developmental research on this topic reveals that children’s strategies for resolving ambiguities significantly differ from those of adults, demonstrating an essential aspect of cognitive development and linguistic interpretation (see [32] for a review). For instance, studies examining referential ambiguities showed that children’s tendency to favour specific cues in resolving referential ambiguities develops at older ages and is less pronounced than in adults [19,33,34,35,36]. Similarly, research on quantifier ambiguities reveals distinct interpretative approaches between children and adults [37,38,39,40,41,42,43,44,45,46], further demonstrating the complexities of cognitive development and linguistic interpretation. Such research dates to Piaget [47,48] (see also [49,50,51]), who highlighted children’s difficulty with the class-inclusion task, a task that involves the understanding that things may belong simultaneously to two categories or classes, one of wider generality and the other narrower (e.g., a dog belongs to the category of dogs and the category of animals).

Building on these findings, more recent research supports the view that children comprehend ambiguous sentences that contain quantifiers differently than adults [37,38,39,40,41,42,43,45,46]. This includes studies that examined children’s comprehension of sentences that contain universal quantificational expressions, such as “every” and “any”, the universal quantifier “every” and negation, the focus particle “only”, and scalar implicatures, such as “some” and “all” [37,39,42,45,46,52,53,54,55]. Several possible explanations have been developed to account for the observed differences between children and adults, such as the view that it is more cognitively costly for children to revise their initial analysis of ambiguous sentences due to their working memory capacity [44,45,56,57] and that children do not always mentally represent a set of alternatives in their discourse model [54]. 

Extending these insights, research into ambiguous sentences containing numerically quantified expressions shows that, while children can access the same interpretations as adults, children have different interpretative preferences [44,58,59,60,61,62,63,64,65]. These differences have been documented in various contexts, including sentences that contain two numerically quantified expressions, such as “Three boys are holding two balloons” [59]; sentences that involve interactions of quantificational expressions with numerically quantified expressions, such as “Two boys pushed a car together” [64]; and numerically quantified expressions and negation, such as “Donald didn’t find two guys” [44,58,59,60,61,62,63,64,65]. Several explanations have been proposed to account for these differences, and the underlying causes of these differences are multifaceted and can be explained by various domains, including semantics, pragmatics, and syntax [59,66,67,68].

It should be clear by now that children use implicit and explicit references differently than adults, implying that the process of discourse comprehension undergoes qualitative changes as children mature. This progression is apparent not only in how anaphoric devices are used to link sections of text but also in how ambiguities are resolved. Nevertheless, for a more thorough understanding of the mechanisms underlying children’s sentence processing system, it is vital to explore how children interpret discourses containing other types of anaphors. Building on previous work on numerically quantified expressions [44,58,59,60,61,62,63,64,65], the present study focused on referential ambiguities that arise when two numerically quantified expressions are used in two-sentence discourses, such as (1):

(1) Three cats were on a wall. Two cats caught a mouse.

The quantified noun phrase “two cats” in (1) is ambiguous, as it can be analysed following two readings. In one reading, it can be analysed as an anaphoric subset reading and interpreted as referring to the set of “three cats” that has already been established as a discourse referent. In other words, there were three cats on the wall, two of which caught a mouse. Alternatively, the quantified noun phrase “two cats” can be analysed as a non-anaphoric new-set reading and interpreted as introducing a new set of referents into the discourse. Following this reading, three cats were on the wall, and two others caught a mouse. A similar ambiguity arises if the quantified noun phrase is replaced with a bare cardinal where the noun is omitted (e.g., “three cats… two…”). Now consider the discourses in (2) and (3); contrary to (1), these are disambiguated in favour of one reading. Unambiguous controls are crucial for understanding how comprehenders process numerically quantified expressions since they enable us to examine comprehenders’ interpretations under unambiguous reading conditions.

(2) Three cats were on a wall. Two *of the* cats caught a mouse.
(3) Three cats were on a wall. Two *other* cats caught a mouse.

In (2), the use of a partitive construction “of the” disambiguates the numerically quantified noun phrase “two cats” in favour of the subset reading. Therefore, the successful comprehension of discourses like (2) depends on the reader’s ability to anaphorically link the elements described in the second sentence with those described in the first sentence. In (3), the determiner “other” disambiguates the quantified expression “two cats” in favour of a new-set reading. Thus, such discourses should be analysed non-anaphorically. 

Experiments with adults using similar materials to the examples shown in 1–3 indicate a strong preference by adults for the subset reading [69,70,71,72]. For instance, in a series of experiments, Frazier et al. [69], using offline methods such as sentence completion and judgement tasks, found that 65% of the time, adults preferred to analyse ambiguous discourses like (1) following a subset reading. Furthermore, an eye-movement study demonstrated longer reading times for new-set readings across ambiguous and unambiguous contexts, a preference corroborated in subsequent studies across German and Korean [69]. Frazier and colleagues [69] attributed this finding to the syntactic principle of Minimal Lowering, which suggests that quantifiers should be maintained in their surface position for processing economy and readers should avoid “lowering” them into new discourse referents [73,74]. Subsequent studies by Kaan and colleagues [70,72] supported adults’ strong subset-reading preference, attributing this to the higher processing costs of new-set readings. This idea is consistent with the Optimality Theoretic Account, which emphasises the role of processing costs in establishing new discourse referents and the Forward Directionality constraint, which suggests a subset reading for ambiguous expressions [75] (see also [76]). Using Event-Related Potentials (ERPs), Kaan et al. [70] confirmed adults’ preference for the subset reading and highlighted the processing difficulties associated with new-set interpretations. They suggested that this finding reflected storage costs associated with new referent introductions [70]. Similarly, using eye-tracking technology, Paterson et al. [71] found similar results and proposed that the processing difficulty emerges late in sentence processing and adults prefer to assign a subset reading to a quantified noun phrase, not because of a preference for assigning a subset reading to an ambiguity but rather because of costs associated with the establishment of new referents.

This body of work collectively illustrates adults’ strong preference for interpreting discourses that contain ambiguous numerically quantified expressions following the subset reading. In the existing developmental literature, to our knowledge, only Wijnen et al. [77] examined children’s interpretations of ambiguities that arise when the discourse contains numerically quantified expressions. Wijnen and colleagues’ study produced striking results, suggesting that, similar to adults, children analyse ambiguous bare cardinals following the subset reading (but see the unpublished studies of [78,79], which did not replicate this finding). Specifically, Wijnen et al. conducted two experiments examining how English- and Dutch-speaking four-year-olds interpret ambiguous discourses that contain bare cardinals. Utilising a variant of the truth-value judgement task [80], the researchers presented the children with stories accompanied by a single picture. The critical sentence containing a bare cardinal was posed as a question, requiring the children to assess with a “yes” or “no” whether the picture accurately depicted the story narrative. An example story is shown in (4).

(4) Here’s a playground. It’s great to do all kinds of funny things when you’re out in the playground, like swinging, making a sand castle or climbing on the monkey bars. There are some kids playing in the sand box.

Are two upside down?

In Wijnen et al.’s [77] study, children were exposed to fifteen ambiguous experimental stories, similar to the example in (4), alongside ten filler stories, divided into three conditions differentiated by accompanying pictures. These depicted scenarios like (a) two children upside down inside the sandbox, corresponding to the subset reading; (b) two children upside down outside the sandbox, corresponding to the new-set reading; and (c) a picture where two adults were upside down outside the sandbox, suggesting a non-anaphoric reading where the bare cardinal “two” is interpreted as two of anything. Their results showed a pronounced preference by English-speaking children for the subset readings (84%), compared to 35.6% for the new-set and 26.7% for the non-anaphoric reading of ambiguous bare cardinals. A strong preference for a subset reading was observed in a follow-up experiment among Dutch-speaking children.

This finding is remarkable, given that previous developmental research showed that children have difficulty using anaphors, like pronouns and the definite reference, to link sentences that refer to previously mentioned entities [11,12,13,14,15,16,17,19,20,21,22,23,24] and that they resolve ambiguities differently to adults [44,58,59,60,61,62,63,64,65]. In contrast, Wijnen et al.’s [77] findings suggest that children have no difficulty with anaphoric bare cardinals, which marks a significant deviation from these challenges. Their results also appear at odds with adult studies [69,70,71,72], where adults displayed less pronounced bias for anaphoric reading than children. 

One possible explanation of Wijnen and colleagues’ [77] findings is the methodology used in their studies. The absence of unambiguous control conditions in their experiments raises questions about the specificity of children’s preferences towards subset readings and suggests the possibility of task-specific strategies influenced by the presentation of only ambiguous scenarios. Wijnen et al. focused exclusively on ambiguous bare cardinals without unambiguous control conditions, raising questions about the interpretive preferences observed in children. This approach contrasts with studies like Frazier et al. [69] and Paterson et al. [71], which included disambiguated discourses, allowing for a clearer understanding of adult interpretations. The reliance on ambiguous stories could lead children to develop task-specific strategies, possibly skewing the results towards subset readings not because of a natural preference but due to the experimental design.

This discrepancy suggests the need to test children’s interpretations against ambiguous and unambiguous contexts to thoroughly examine their interpretations of numerically quantified expressions and accurately compare them to adults’ comprehension. Without incorporating unambiguous scenarios, conclusions about children’s preference for subset readings remain speculative, limiting our understanding of their interpretative preferences compared to adults. To address this issue, we conducted six experiments to examine children’s interpretations of ambiguous and unambiguous numerically quantified discourses. Our general objective was to determine whether children, like adults, tend to interpret numerically quantified expressions with an anaphoric subset reading or opt for a non-anaphoric new-set reading. Across a series of experiments, adult and child participants were presented with pictures and asked to select the one they believed corresponded to the discourse’s meaning, choosing between images representing either a new-set or a subset reading of the numerically quantified expression. A preference for the subset reading would indicate an anaphoric interpretation of numerically quantified expressions akin to other anaphor types. Conversely, a preference for the new-set reading would suggest a different cognitive processing strategy by the participants. We expected skilled adult readers to select the appropriate picture for discourses containing an unambiguous expression and to preferentially select the picture depicting a subset reading for discourses containing an ambiguous expression, while the experiments would reveal whether children exhibited the same preferences.

## 2. General Materials and Methods

The six experiments reported in this paper used similar materials and methods; thus, this section provides the common elements.

### 2.1. Sample

Participants were native Greek-speaking children and adults from Cyprus. The adult participants comprised 50 postgraduate students, while the child participants consisted of 249 typically developing first- and second-graders aged 6–8 years, recruited from four primary schools. None of the child participants were known to have a learning disability. A different set of participants were recruited for each of the six experiments. The research received the necessary ethical approval from the University of Leicester’s School of Psychology Research Ethics Committee and Cyprus’s Ministry of Education, and all research procedures were performed in accordance with their guidelines and regulations. Adult participants were recruited from different universities in Cyprus, whereas, for child participants, the experimenter approached local primary schools, explained the study’s objectives, and asked for the headteachers’ consent for the study to be conducted in their schools. Upon receiving the headteachers’ approval, the experimenter distributed letters to parents and guardians of first- and second-grade children and requested their written consent for their children’s participation. This approach ensured that all participating children had the appropriate parental permission. Anonymity was assured for all participants. Additionally, prior to taking part in the experiment, children were informed that participation was voluntary and provided their verbal consent. As a token of appreciation for taking part, children were given a pencil as a gift at the end of the experiment.

### 2.2. Materials and Design

#### 2.2.1. Introductory Phase

Basic demographic information, including age, gender, and native language, was collected from the participants. Additionally, given the focus on numerical expressions in this study, ensuring that the child participants had basic competence in mathematics was essential. Therefore, before the experimental phase, children were assessed using three simple arithmetic tasks: (a) counting nine balls and stating the total, (b) solving three basic addition problems (i.e., 5 + 3, 4 + 2, and 3 + 2), and (c) completing three simple subtraction problems (i.e., 5–3, 4–2, and 3–2). Failure to correctly answer any of these tasks indicated potential difficulties in participating in the comprehension experiment. Consequently, only the responses of children who achieved 100% accuracy on these preparatory tasks were included in subsequent analyses. There was no introductory phase for the adult participants.

#### 2.2.2. Experimental Phase

This study investigated how participants interpret referential ambiguities arising from numerically quantified noun phrases (e.g., “two cats”) and bare cardinals (e.g., “two”) presented in short two-sentence discourses across three experimental conditions. Examples of the three experimental conditions are shown in 1–3, repeated here as 5–7.

(5) Three cats were on a wall. (Two cats/Two) caught a mouse. (Ambiguous)
(6) Three cats were on a wall. Two (of the cats/of them) caught a mouse. (Unambiguously subset)
(7) Three cats were on a wall. Two (other cats/others) caught a mouse. (Unambiguously new-set)

In each scenario, the initial sentence introduced a potential referent (e.g., three cats), while the subsequent sentence used either an ambiguous reference (“two cats” or “two”), a partitive expression (“two of the cats” or “two of them”), or a determiner (“two other cats” or “two others”) to denote entities within the same category. The ambiguous numerically quantified expression could always refer to either a subset of the initially mentioned referents (e.g., two of the three cats) or an entirely new set of referents (e.g., two other cats). In contrast, the partitive expression unambiguously referred to a subset reading, and the determiner unambiguously indicated a new set of referents. In total, participants were presented with 15 experimental short discourses, 5 for each type (i.e., 5 ambiguous, 5 unambiguously subset, and 5 unambiguously new-set).

**Figure 1:**
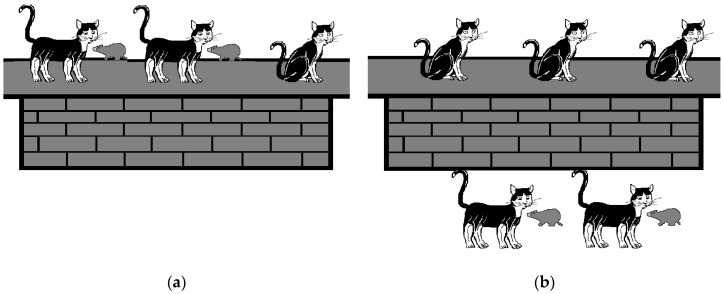
(**a**) An example picture depicting the subset reading of the short discourse; (**b**) an example picture showing the new-set reading of the short discourse.

Each short discourse was accompanied by a series of pictures illustrating the possible interpretations, printed in black and white on A3 paper, with the order of presentation randomised by the experimenter. In Experiment 1, participants were presented with two pictures with the task of selecting the one that corresponds to the discourse meaning. Figure 1a illustrated a subset interpretation, where, according to the scenarios in Examples 5–7, two of the three cats on the wall caught a mouse, resulting in a total depiction of three cats. Figure 1b presented a new-set reading, displaying three cats on the wall but two additional cats catching a mouse, totalling five cats. Figure 1 provides examples of the pictures used alongside the short discourses (5)–(7).

**Figure 2:**
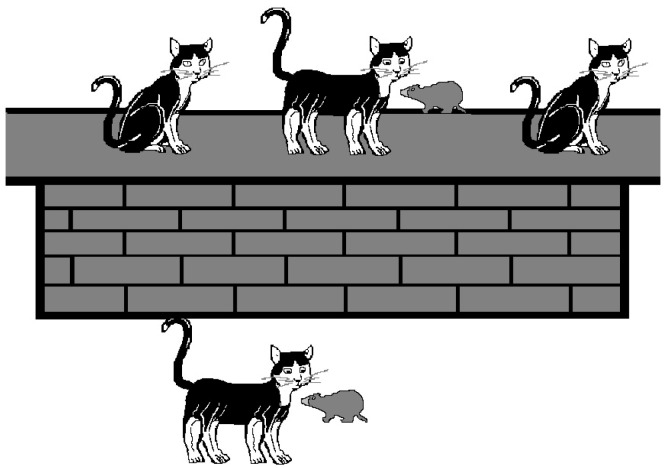
An example of a picture depicting a non-integrative reading of the short discourse.

To explore whether participants could conceptualise discourses without adopting a subset or new-set interpretation, Experiment 2 introduced a third, non-integrative picture, where participants had to again choose the one they believed best corresponded to the short discourse. The non-integrative picture was designed to allow the independent analysis of each sentence, not fitting neatly into either subset or new-set interpretations. For instance, for the short discourses in (5)–(7), the non-integrative picture showed three cats on the wall and two with a mouse, but the total number of cats does not align with either the subset (three cats) or new-set (five cats) interpretation. This picture was used in Experiments 2–6. Figure 2 shows an example of the non-integrative picture used alongside the short discourses (5)–(7).

**Figure 3:**
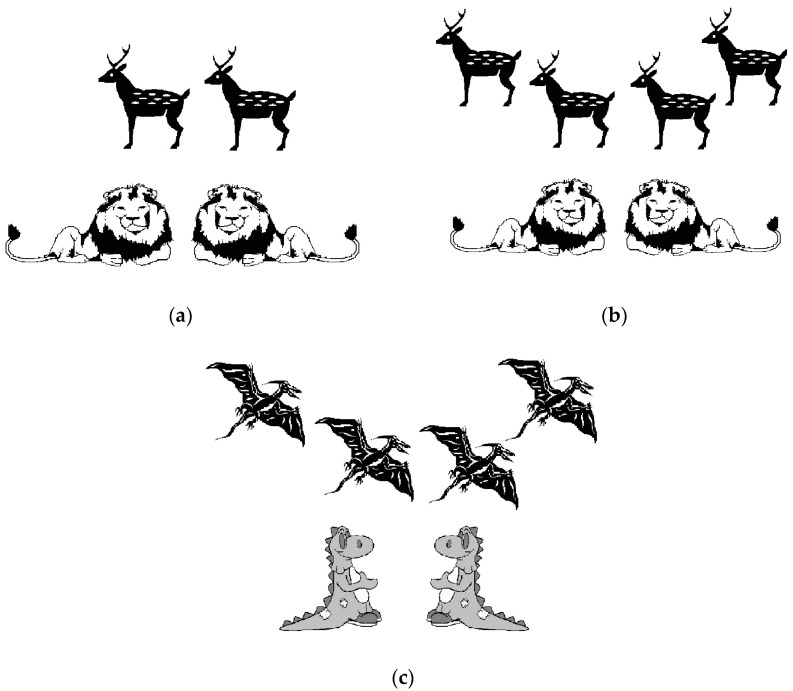
(**a**) An example picture depicting the correct type of entities but an incorrect number; (**b**) an example picture showing both the correct type and correct number of entities; (**c**) a distractor picture displaying entities of the wrong type.

The 15 experimental stimuli were mixed with 5 fillers, in which the first and second sentences referenced different sets of entities (e.g., “four deer… two lions…”), making them unsuitable for a subset or a new-set interpretation. From Experiment 2 onwards, a distractor picture was also presented so that the fillers would have the same number of pictures as the experimental materials. The distractor picture depicted entities different from those mentioned in the discourse. Notably, these filler items served to assess participants’ attentiveness to the task. Responses from participants who failed to correctly identify the appropriate picture for the fillers were excluded from subsequent analyses, and only the responses of participants who understood and paid attention to the task were analysed. Figure 3 shows examples of the filler items for the short discourse presented in (8).

(8) Four deer were in the forest. Two lions were resting.

To ensure participants’ responses reflected their interpretative preferences rather than being influenced by a specific version of the short discourse, each experimental discourse was presented in three formats: ambiguous, unambiguously subset, and unambiguously new-set, to different groups of participants. Consequently, three material files were prepared, each containing a different version of each experimental discourse. For instance, the “cat example” (refer to short discourses in Examples 5–7) was presented as unambiguously new-set in the first file, ambiguous in the second, and unambiguously subset in the third. Participants were exposed to only one material file, ensuring they encountered no more than one version of each experimental discourse. The same five filler items were included in all material files. Each file contained an equal distribution of ambiguous, unambiguously new-set, unambiguously subset, and filler two-sentence discourses. Furthermore, to ensure that the order of presentation did not bias participants towards a particular response pattern, half of the participants received the discourses in a fixed sequence, while this sequence was reversed for the other half.

### 2.3. Procedure

Children were individually tested in a quiet room within their school, while adult participants were tested in a quiet area of their college, with each session lasting around 20 min. For children, the session began with an introductory phase, where they were administered the arithmetic tasks, and only those who correctly solved all of the tasks advanced to the experimental phase. At the beginning of each trial, the experimenter read the short discourse aloud (without stressing any words) and then presented the corresponding pictures. The same procedure was used with adult participants, except they commenced directly with the experimental phase.

### 2.4. Analysis

Responses from participants who did not choose the correct picture for all five filler items were excluded from the analyses. Data for all six experiments were analysed using mixed-design ANOVAs, both by participants (F_1_) and by items (F_2_).

## 3. Experiments

### 3.1. Experiment 1

#### 3.1.1. Objective

The first experiment had two primary objectives: (a) to examine the interpretative preferences of adults and children when presented with ambiguous and unambiguous discourses containing numerically quantified expressions and (b) to explore potential differences in the interpretation of discourses containing bare cardinals as opposed to those with quantified noun phrases. To address the second objective, participants were divided into two groups. One group was presented with both ambiguous and unambiguous discourses featuring numerically quantified noun phrases (e.g., “three cats… two cats…”), while the other group was presented with discourses containing bare cardinals (e.g., “three cats… two…”). Based on the objectives and the existing literature, we formulated the following hypotheses: 

First, adults should strongly prefer the anaphoric subset reading for ambiguous discourses and provide the expected interpretation for unambiguous discourses. Thus, adults are expected to choose the picture depicting a subset interpretation when interpreting either the ambiguous or unambiguous subset discourse and the picture depicting a new-set reading when interpreting the unambiguously new-set discourse. 

We also expected that children would produce the expected interpretations of the unambiguous discourses. That is, they should select the “subset picture” for unambiguously subset discourses and the “new-set picture” for unambiguously new-set discourses. We considered the critical question to be whether the children would exhibit the same preference for assigning a subset reading to the ambiguous discourses as adults do and so preferentially select the “subset picture”. Alternatively, if they have difficulty forming the necessary referential links to support a subset reading of the ambiguity during comprehension, then, in this case, they may prefer to assign a new-set reading to the ambiguity and may prefer to select the “new-set picture” in response to an ambiguous discourse. Finally, it was of interest to determine whether the two age groups assign similar interpretations to bare cardinals (e.g., “two”) as they do to quantified nouns (e.g., “two mice”).

#### 3.1.2. Sample

Sixty-eight children and forty adults participated in this experiment. The participants were divided into two groups. The Quantified Noun Phrase Group included 34 first-graders with a mean age of seven years and three months, alongside 20 adults. This group was presented with both ambiguous and unambiguous discourses containing numerically quantified noun phrases. The Bare Cardinal Group comprised 34 second-graders with a mean age of eight years and three months and 20 adults, who were presented with ambiguous and unambiguous discourses containing numerically quantified bare cardinals.

#### 3.1.3. Materials and Design

This experiment employed a 2-alternative forced-choice picture selection task. Participants were presented with 20 short discourses together with two pictures: one reflecting the subset interpretation of the discourse and the other the new-set interpretation (see Figure 1 for an example) with the task of selecting the picture that best matched the meaning of the discourse.

#### 3.1.4. Results and Discussion

Before analysing the data, participants’ responses were checked, and those who failed the arithmetic problems and/or filler items were excluded from further analyses. Among the adult participants, none failed the filler items. However, of the 68 children who participated, 15 were excluded due to failure in the arithmetic tasks, the incorrect selection of pictures for the filler items, or both. Consequently, the final number of analysed responses comprised 40 adults and 53 children (mean age seven years and eight months). Of these participants, 25 children and 20 adults composed the Quantified Noun Phrase Group, and 28 children and 20 adults belonged to the Bare Cardinal Group.

The frequency with which children and adults selected the “new-set picture” was analysed using two 2 (age: children or adults) × 3 (discourse type: unambiguously new-set, ambiguous, and unambiguously subset) × 2 (numerically quantified expression: quantified noun phrase or bare cardinal) mixed-design ANOVAs. 

The first concern was to establish whether the two age groups assigned similar or different interpretations of the discourses including quantified noun phrases or bare cardinals. The analysis revealed that, although there was a statistically significant effect of discourse type (*F*_1_(2, 178) = 199.76, *p* < 0.05, *η*^2^_p_ = 0.692; *F*_2_(2, 112) = 223.31, *p* < 0.05, *η*^2^_p_ = 0.800) and a statistically significant difference between discourse type and age group (*F*_1_(2, 178) = 141.34, *p* < 0.05, *η*^2^_p_ = 0.614; *F*_2_(2, 112) = 152.33, *p* < 0.05, *η*^2^_p_ = 0.731), there was no significant difference in the way that participants treated the two types of numerically quantified expressions (*F*_1_(2, 178) = 1.13, *p* > 0.05, *η*^2^_p_ = 0.013; *F*_2_(2, 112) = 0.39, *p* > 0.05, *η*^2^_p_ = 0.007). Moreover, the results showed no three-way interaction between discourse type, numerically quantified expression, and age group (*F*_1_(2, 178) = 0.79, *p* > 0.05, *η*^2^_p_ = 0.009; *F*_2_(2, 112) = 0.58, *p* > 0.05, *η*^2^_p_ = 0.010), indicating that the type of numerically quantified expression did not interact with any other variables. Therefore, the analysis provided no evidence that participants interpreted the discourses containing a quantified noun phrase differently from those containing a bare cardinal. As it seemed unnecessary to conduct separate analyses across these two conditions, the responses for the Quantified Noun Phrase Group and the Bare Cardinal Group were combined.

**Figure 4:**
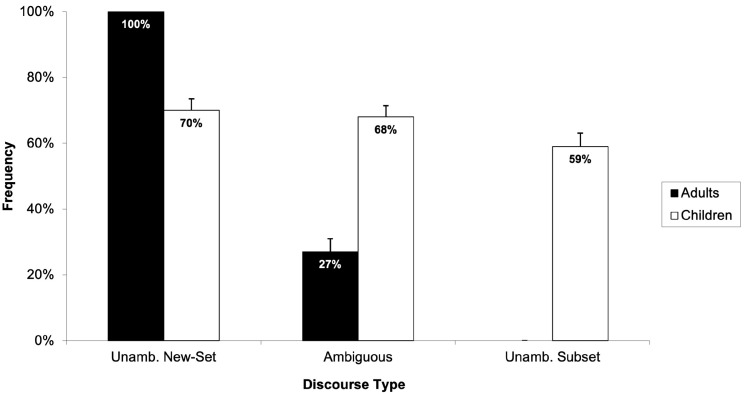
The frequency with which children and adults selected the picture depicting a new-set reading in Experiment 1. Error bars represent the standard error of the mean.

Subsequent analyses focused on age differences in the interpretations given to the ambiguous and unambiguous discourses. Figure 4 illustrates the frequency with which children and adults selected the picture depicting a new-set reading in the three discourse types collapsed across the two types of numerically quantified expressions (i.e., quantified noun phrase and bare cardinal). 

A two 2 (age group: children and adults) × 3 (discourse type: unambiguously new-set, ambiguous, and unambiguously subset) mixed-design ANOVAs showed a statistically significant main effect of discourse type (*F*_1_(2, 182) = 201.19, *p* < 0.05, *η*^2^_p_ = 0.689; *F*_2_(2, 116 = 227.34, *p* < 0.05, *η*^2^_p_ = 0.797) and a statistically significant interaction between discourse type and age group (*F*_1_(2, 182) = 140.275, *p* < 0.05, *η*^2^_p_ = 0.607; *F*_2_ (2, 116) = 155.071, *p* < 0.05, *η*^2^_p_ = 0.728). Subsequent post hoc tests using Tukey’s HSD were computed to further explore the responses of children and adults across the three discourse types. For adults, post hoc analysis indicated statistically significant differences in the frequency of selecting the “new-set picture” across all three discourse types. Specifically, adults selected the “new-set picture” significantly more often for the unambiguously new-set discourse type than for both the unambiguously subset (*p* < 0.05) and ambiguous discourse types (*p* < 0.05) and more frequently for the ambiguous than the unambiguously subset discourse type (*p* < 0.05). In contrast, the post hoc analysis revealed that children selected the “new-set picture” statistically significantly more often for the unambiguously new-set discourse type compared to the unambiguously subset discourse type (*p* < 0.05) and similarly for the ambiguous compared to the unambiguously subset discourse type (*p* < 0.05). However, no statistically significant difference was observed in the frequency of children selecting the “new-set picture” between the unambiguously new-set and ambiguous discourses (*p* > 0.05). Strikingly, the results suggest that children treated ambiguous numerically quantified expressions and those explicitly marked for a new-set reading in a comparable manner. 

To ascertain whether participants’ responses differed significantly from chance, one-sample *t*-tests were computed for each discourse type (i.e., unambiguously new-set, ambiguous, and unambiguously subset). The *t*-tests compared the participants’ mean against a test value of 50%. Since adults’ frequency of selecting the “new-set picture” across the three discourse types was very clear (i.e., 100% for unambiguously new-set discourse type, 27% for ambiguous discourse type, and 0% for unambiguously subset discourse type), *t*-tests were computed only for children’s responses. The results of the *t*-test analysis showed that children’s responses were statistically significantly different to the 50% chance level across all discourse types: (*t*(52) = 5.63, *p* < 0.05) for the unambiguously new-set, (*t*(52) = 5.28, *p* < 0.05) for the ambiguous, and (*t*(52) = 2.15, *p* < 0.05) for the unambiguously subset discourse types. These findings suggest that children’s preference for the “new-set picture” in interpreting ambiguous and unambiguous numerically quantified expressions was not attributable to chance but rather a systematic preference.

Experiment 1 showed that adults strongly prefer the anaphoric subset reading in ambiguous numerically quantified expressions and accurately select the corresponding picture in unambiguous conditions. This finding is in accordance with previous studies with adults [69,70,71,72]. Additionally, there was clear evidence for differences in the interpretative preferences of children and adults. Specifically, children selected the “subset picture” as correctly corresponding to ambiguous numerically quantified expressions only 32% of the time. Moreover, contrary to our hypothesis, children did not consistently produce the anticipated interpretation for unambiguous discourses. Significantly, our results showed that, unlike adults, six- to eight-year-old children demonstrated a pronounced preference for the “new-set picture” across both ambiguous and unambiguous contexts. This finding is particularly striking for the unambiguously subset discourses, where the partitive construction “of the” failed to guide children towards a subset interpretation. Taken together, the children’s responses challenge the limited existing research on children’s interpretative strategies when analysing numerically quantified expressions [77], suggesting a more complex process of discourse interpretation among children.

Aligned with prior research on anaphors, quantifiers, and ambiguities, we propose two potential explanations for children’s preferences. One possibility is that the “new-set picture” preference may reflect children’s broader difficulty with anaphors as tools for text cohesion, not necessarily a preference for a new-set reading. This challenge might arise from (i) difficulties in reaching the anaphoric subset reading, (ii) the subset reading not being readily available to them, (iii) integration difficulty that leads to separate analyses of the two sentences, or (iv) a cognitive cost of integration, prompting a choice for the “new-set picture” that aligns with a non-integrative interpretation. Consequently, children’s pronounced selection of the “new-set picture” in the present experiment could stem from obstacles in processing anaphoric subset readings rather than a preference for new-set interpretations of numerically quantified expressions. Another possibility is that the variance found in this experiment might indicate inherent differences in interpretative strategies between children and adults. Following this suggestion, children’s selection of the “new-set picture” over the “subset picture” reflects a preference for the new-set interpretation. That is, children acknowledge both potential readings but, when forced to choose, opt for their preferred interpretation. This suggests that if children are allowed to evaluate both images against the discourse, they might indicate both as valid interpretations. These possibilities will be explored further in the following experiments.

### 3.2. Experiment 2

#### 3.2.1. Objective

Experiment 2 aimed to investigate whether children’s preference for the picture depicting a new-set reading observed in Experiment 1 was due to integration difficulties, leading them to favour a non-integrative interpretation. To explore this, an additional “non-integrative picture” was introduced. This was designed to independently satisfy the conditions of both sentences without presenting an integrative meaning, thereby conflicting with both new-set and subset interpretations (see Figure 2 for an example).

Considering that adults demonstrate a strong preference for the subset interpretation of ambiguous quantified expressions, in this experiment, they served as a control group to validate the assumption that the “non-integrative picture” would be less likely chosen by those without integration difficulties. For children, introducing the “non-integrative picture” allowed us to assess whether their previous selections were influenced by challenges in integrating discourse components.

As in Experiment 1, we expected adults to exhibit a strong preference for the “subset picture” that aligns with an integrated interpretation of ambiguous quantified expressions and to select the appropriate picture for discourses that have an unambiguous subset or new-set reading. Adults were also expected to consistently disregard the “non-integrative picture” across all discourse types (i.e., unambiguously new-set, ambiguous, and unambiguously subset), confirming their capability for integration.

By comparison, we considered whether the presence of a “non-integrative picture” might influence children’s selection patterns. A strong preference for the “non-integrative picture” or a balanced selection between the “non-integrative picture” and the “new-set picture” might provide evidence that the effects observed in Experiment 1 were due to integration difficulties. Similarly, a preference for selecting the “non-integrative picture” or a balanced selection between the “non-integrative picture” and “new-set picture” for discourses with an unambiguously subset reading might suggest that children also had difficulty integrating sentences in these discourses.

#### 3.2.2. Sample

Fifty-one first- and second-grade children with a mean age of seven years and four months, along with ten adults, participated in this experiment.

#### 3.2.3. Materials and Design

In Experiment 2, we used the same discourses as in Experiment 1. However, given the lack of significant differences in participants’ interpretations of discourses featuring bare cardinals versus quantified noun phrases in Experiment 1, Experiment 2 focused exclusively on discourses containing numerically quantified noun phrases. Participants engaged in a 3-alternative forced-choice picture selection task, where they chose the picture that most accurately represented the discourse’s meaning. The options included the two pictures used in Experiment 1 (see Figure 1 for an example) and a picture representing a non-integrative interpretation (refer to Figure 2 for an example). Children were also presented with a third picture for the filler items to ensure consistency across the materials. This additional picture, serving as a distractor, was unrelated to the discourse content (see Figure 3 for an example).

#### 3.2.4. Results and Discussion

**Figure 5:**
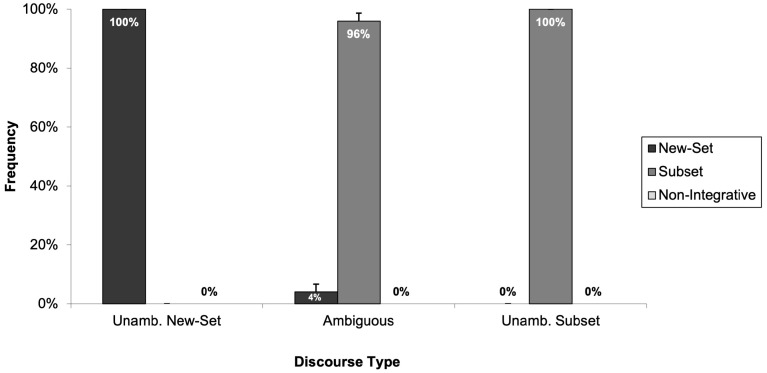
The frequency with which adults selected the three pictures in Experiment 2, with error bars representing the standard error of the mean.

First, consider the data for the adult participants. As illustrated in Figure 5, adults’ selections aligned with our predictions; adults never identified the “non-integrative picture” as an accurate description of the discourses. This supported our hypothesis that the “non-integrative picture” would unlikely be chosen by readers who can integrate sentences effectively. Rather, as in Experiment 1, adults selected the appropriate “new-set picture” and “subset picture” for the unambiguous discourses and showed an overwhelming preference for selecting the “subset picture” for ambiguous discourses.

**Figure 6:**
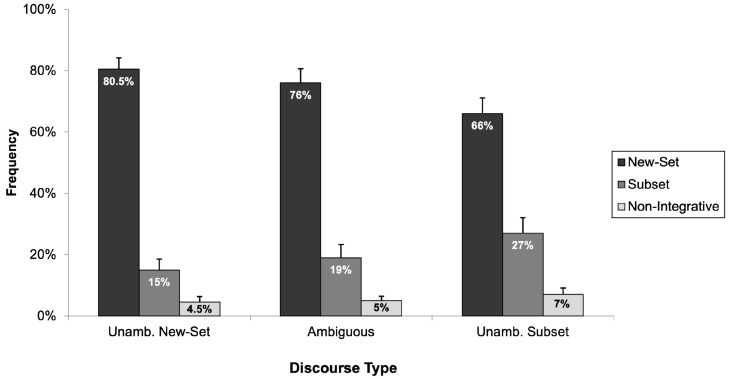
The frequency with which children selected the three pictures in Experiment 2, with error bars representing the standard error of the mean.

Now consider the children’s data. Introducing the “non-integrative picture” had little influence on the children’s response patterns. Rather, the children continued to show a strong preference for selecting the “new-set picture” regardless of whether the discourses were ambiguous or had an unambiguous “new-set” or “subset” reading. Figure 6 depicts the frequency with which children selected each of the three pictures (“new-set picture”, “subset picture”, and “non-integrative picture”) across the three discourse types. 

The analysis excluded responses from 12 children who did not successfully complete the fillers and/or arithmetic task, leaving data from 39 children with a mean age of seven years and four months for evaluation. The frequency with which children selected the picture depicting a new-set interpretation in all three discourse types (i.e., unambiguously new-set, ambiguous, and unambiguously subset) was analysed using two 3 (discourse type: unambiguously new-set, ambiguous, and unambiguously subset) × 1 (selection of the “new-set picture”) mixed-design ANOVAs. The analysis revealed a statistically significant main effect of discourse type (*F*_1_(2, 76) = 4.97, *p* < 0.05, *η*^2^_p_ = 0.116; *F*_2_(2, 28) = 6.40, *p* < 0.05, *η*^2^_p_ = 0.313). Post hoc tests using Tukey’s HSD showed significant differences in the frequency of “new-set picture” selections between the unambiguously new-set and unambiguously subset discourse types (*p* < 0.05), with children selecting the “new-set picture” significantly more frequently in the unambiguously new-set discourse type than in the unambiguously subset discourse type. Critically, like in Experiment 1, there was no significant difference in the frequency of selecting the “new-set picture” between unambiguously new-set and ambiguous discourse types (*p* > 0.05), indicating a consistent response pattern among children for these discourse types. Lastly, there were no significant differences in selection frequency between the ambiguous and unambiguously subset discourse types (*p* > 0.05). Therefore, in comparison with Experiment 1, children’s “new-set picture” selection was similar for ambiguous and unambiguously subset discourse types. A potential explanation for this outcome is the third picture choice, which may have influenced their responses.

The results broadly align with the pattern in Experiment 1, demonstrating that six- to eight-year-old children exhibit a strong preference for the “new-set picture” across both ambiguous and unambiguous numerically quantified expressions. We also found that children did not perceive the “non-integrative picture” as an accurate representation of the discourse in any discourse type.

Children’s preference for the “new-set picture” is very clear, but it was essential to examine the selection patterns against a 50% chance level. This analysis aimed to determine whether children’s choices exhibited any systematic preference beyond the “new-set picture”. Therefore, we removed children’s preference for the “new-set picture” and computed one-sample *t*-tests to examine whether children’s selection between the “subset picture” and the “non-integrative picture” was significantly different from the 50% chance level. For the unambiguously new-set discourse type, *t*-tests revealed that children’s selections between the “subset picture” and the “non-integrative picture” did not statistically significantly differ from the 50% chance level (*t*(20) = 1.90, *p* > 0.05), suggesting no strong preference for these options when the discourse was disambiguated towards a new-set reading. However, in ambiguous and unambiguously subset discourse types, analyses indicated statistically significant deviations from chance: (*t*(20) = 2.39, *p* < 0.05) for ambiguous discourse types and (*t*(26) = 3.37, *p* < 0.05) for unambiguously subset discourse types. These results imply that when children did not select the “new-set picture”, they had a preference for the “subset picture” over the “non-integrative picture”.

Taken together, Experiment 2 replicated the finding that children have a strong preference for the “new-set picture” even though they had an additional non-integrative option. This finding challenges the effectiveness of the “non-integrative picture” in assessing integration difficulties. Nonetheless, subsequent experiments will continue incorporating the “non-integrative picture” to explore whether task modifications could potentially alter children’s preferences.

### 3.3. Experiment 3

#### 3.3.1. Objective

The aim of Experiment 3 was to examine whether the strong preference for the “new-set picture” was influenced by an arithmetic strategy prompted by the use of two numerical expressions within the discourse (e.g., “three cats… two cats…”). Adding the two numerals could explain children’s strong preference for the “new-set picture” since this picture always represented the total of entities. Inevitably, therefore, the “new-set picture” could also be selected if children followed an arithmetic strategy. Note also that this arithmetic strategy could be prompted by our tests of arithmetic competence prior to the experiment. To address this potential confound, Experiment 3 modified the numerically quantified noun phrase in the first sentence to a natural language quantifier (e.g., “three cats… two cats…” was changed to “some cats… two cats…”) to assess children’s selection patterns under these new conditions. This adjustment aimed to discern whether the children’s preferences were attributable to a counting strategy or rooted in a more fundamental aspect of language comprehension. Based on this objective, we investigated whether children continued to exhibit a strong preference for the “new-set” reading when the quantified noun phrases were modified to discourage an arithmetic strategy.

#### 3.3.2. Sample

The participants were 38 children, with a mean age of seven years and four months. 

#### 3.3.3. Materials and Design

In Experiment 3, the materials from the fifteen experimental discourses underwent modification (while retaining the same five fillers as in Experiment 2). Specifically, we replaced the numerical quantifier in the first sentence of each discourse with one of three alternatives: (i) the natural language quantifier “some”, (ii) the natural language quantifier “many”, or (iii) the collective noun phrase “a group of”, with each type being evenly distributed among the fifteen experimental discourses. Given that “many” and “a group of” imply a higher number of entities, the corresponding pictures for discourses with these phrases were adjusted to depict an increased number of entities. Conversely, pictures for discourses utilising the natural language quantifier “some” remained unchanged from those used in previous experiments.

#### 3.3.4. Results and Discussion

**Figure 7:**
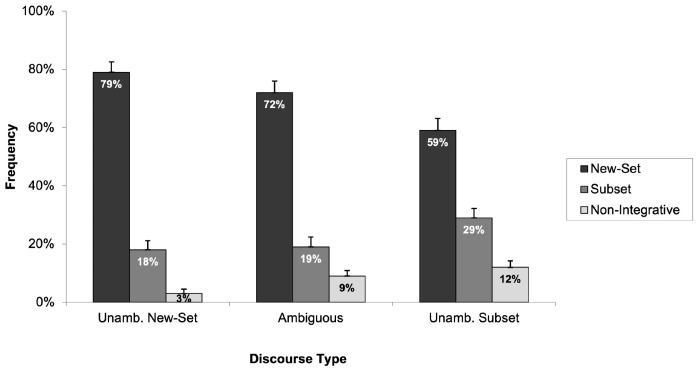
The frequency with which children selected the three pictures in Experiment 3, with error bars representing the standard error of the mean.

From the 38 children who participated in this experiment, responses from two children were excluded due to their failure on the arithmetic problems and/or filler items. Consequently, the analysis was conducted on the data from 36 children with a mean age of seven years and three months. Figure 7 presents the percentage of times children selected the “new-set picture”, the “subset picture”, and the “non-integrative picture” across the three discourse types (i.e., unambiguously new-set, ambiguous, and unambiguously subset). As is clearly illustrated in Figure 7, children strongly preferred the picture depicting a new-set reading across all three discourse types despite modification to the discourses to remove the usefulness of an arithmetic strategy. 

The analysis examined the frequency with which children selected the picture depicting a new-set reading across the three discourse types, employing two 3 (discourse type: unambiguously new-set, ambiguous, and unambiguously subset) × 1 (selection of the “new-set picture”) mixed-design ANOVAs. This revealed a significant main effect of discourse type (*F*_1_(2, 70) = 7.68, *p* < 0.05, *η*^2^_p_ = 0.180; *F*_2_(2, 28) = 3.86, *p* < 0.05, *η*^2^_p_ = 0.216), indicating a marked preference for the “new-set picture” in the unambiguously new-set discourse type. Further, Tukey HSD post hoc analysis showed significant differences in the selection frequencies for the “new-set picture” between the two unambiguous discourse types (*p* < 0.05), with a stronger preference shown for the unambiguously new-set discourse type. Additional post hoc comparisons between responses to ambiguous and unambiguously subset discourse types revealed significant differences in children’s frequency of selecting the “new-set picture” (*p* < 0.05), with a preference for the ambiguous over the unambiguously subset discourse type. Consistent with previous experiments, post hoc analysis showed no significant differences in the frequency of selection for the “new-set picture” between unambiguously new-set and ambiguous discourse types (*p* > 0.05), indicating a consistent selection pattern for these types.

We next computed one-sample *t*-tests to assess whether children’s selections of the “subset picture” and the “non-integrative picture” significantly differed from the 50% chance level after excluding the cases where children selected the “new-set picture”. The *t*-test analysis showed that children’s selection between the “subset picture” and the “non-integrative picture” in all three discourse types were significantly different to the 50% chance level: (*t*(21) = 6.09, *p* < 0.05) for the unambiguously new-set discourse type, (*t*(26) = 2.15, *p* < 0.05) for the ambiguous discourse type, and (*t*(31) = 5.08, *p* < 0.05) for the unambiguously subset discourse type. 

The results from Experiment 3 replicate the findings of our earlier experiments. Clearly, six- to eight-year-old children’s preference for the “new-set picture” does not stem from an arithmetic strategy. This preference persisted even after we replaced numerically quantified expressions with non-numerical quantifiers. This finding further supports the notion that the non-adult-like responses observed among children might reflect their underlying language comprehension processes. The experiment also demonstrated that removing the numerically quantified noun phrase did not shift children’s choices towards the “non-integrative picture”. Similarly to what was observed in Experiment 2, only a few children opted for this choice. This experiment also showed a distinct pattern in how children chose the “new-set picture” between ambiguous and unambiguously subset discourse types, diverging from the findings of Experiment 2. This difference might be attributed to the increased selection of the “non-integrative picture” in this experiment. Moreover, when children did not choose the “new-set picture”, they showed a clear preference for the “subset picture” over the “non-integrative picture”, indicating a nuanced understanding of the discourses. 

### 3.4. Experiment 4

#### 3.4.1. Objective

Experiment 4 investigated children’s interpretive processes further. Notably, Experiments 1–3 limited children to selecting only one picture, leaving unresolved whether they might also consider the “subset picture” as an accurate representation of the discourse. To address this, Experiment 4 employed a variation of the forced-choice picture selection task that permits children to select more than one picture. This allowed a more direct examination of whether the strong preference for the “new-set picture” arises from (i) an interpretation following a new-set reading, (ii) a lack of acknowledgement for the subset reading, or (iii) difficulties in integrating succeeding sentences. Additionally, this modification allowed us to examine whether the effects in the previous experiments were influenced by the original task design, which may have unintentionally biased selections towards the “new-set picture”. Lastly, to explore the potential impact of the instruction on children’s selections, Experiment 4 was divided into two versions. In Experiment 4A, children were instructed to select all pictures they considered accurate descriptions of the discourse, and in Experiment 4B, children had to identify any pictures they believed did not correspond to the discourse. The critical question here was whether children would still almost exclusively select (in Experiment 4A) or exclusively not select (in Experiment 4B) the “new-set picture” when presented with the different discourse types. In particular, it was of interest to discover whether they considered either the “subset picture” or the “non-integrative picture” to be a possible depiction of events described in the discourses.

#### 3.4.2. Sample

Thirty-six children participated in this experiment. Fourteen children, with a mean age of seven years and three months, participated in Experiment 4A, and 22 children, with a mean age of seven years and one month, participated in Experiment 4B. 

#### 3.4.3. Materials and Design

The findings from Experiment 3 showed that children’s preference for the “new-set picture” remained unchanged despite removing the numerically quantified expression from the first sentence. Consequently, Experiment 4 used the same discourses and pictures used in Experiment 2 to investigate children’s interpretive preferences under consistent conditions.

#### 3.4.4. Procedure

The procedure followed that of the previous experiments, with a key modification: instead of being restricted to choosing a single picture that best represented the discourse, children were now allowed and encouraged to select multiple pictures if they felt that more than one matched the discourse’s meaning. To ensure clarity in this new approach, the experimenter explicitly reminded the children that multiple selections were possible. In Experiment 4A, participants were instructed to select all pictures that correctly depicted the discourse. Conversely, in Experiment 4B, they were asked to identify all pictures that did not correspond to the meaning of the discourse.

#### 3.4.5. Results and Discussion

**Figure 8:**
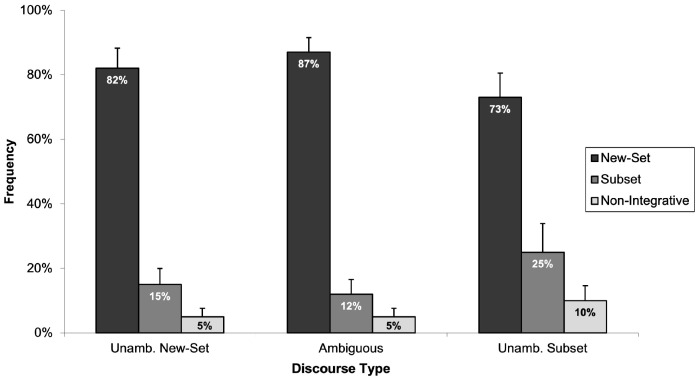
The frequency with which children selected the three pictures in Experiment 4A, with error bars representing the standard error of the mean. Note that children could select multiple pictures.

Analyses were conducted separately for Experiments 4A and 4B. In Experiment 4A, 2 of the 14 participating children were excluded from the analysis due to incorrect responses to the filler items. Consequently, data from 12 children with a mean age of seven years and three months were included in the final analysis. Figure 8 displays the frequency with which children selected the “new-set picture”, the “subset picture”, and the “non-integrative picture” across the three discourse types. 

As is clear from Figure 8, children predominantly chose the “new-set picture” despite the opportunity to select multiple pictures. A closer examination of our data showed that only 4 out of the 12 children selected multiple pictures. As with our previous experiments, we analysed the frequencies with which children selected the “new-set picture” across the three discourse types. Data were analysed using two 3 (discourse type: unambiguously new-set, ambiguous, and unambiguously subset) × 1 (selection of the “new-set picture”) mixed-design ANOVAs. The analysis revealed that there was no significant main effect of discourse type (*F*_1_(2, 22) = 1.37, *p* > 0.05, *η*^2^_p_ = 0.110; *F*_2_(2, 28) = 2.49, *p* > 0.05, *η*^2^_p_ = 0.151), indicating that children selected the “new-set picture” with similar frequency across different discourse types. This finding suggests that the discourse type did not significantly influence children’s preferences for the “new-set picture”. A potential explanation for the lack of significant findings could be the small sample size in this experiment, which may have limited the statistical power necessary to detect significant effects.

**Figure 9:**
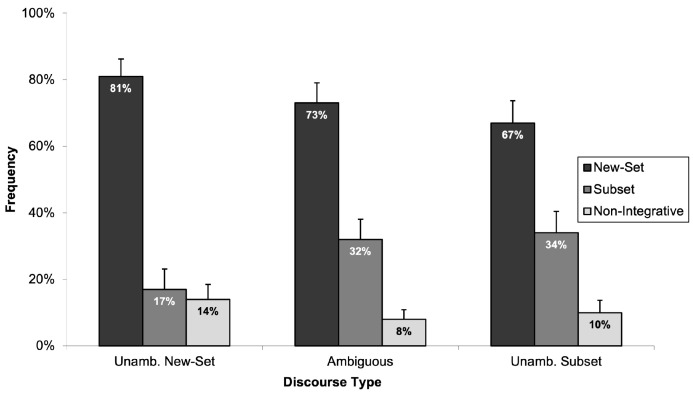
The frequency with which children did not select each picture in Experiment 4B, with error bars representing the standard error of the mean. Note that children could select multiple pictures.

We then examined the responses of children in Experiment 4B. As in this version of the experiment, children were asked to indicate the pictures that did not correspond to the meaning of the discourse, for the results to be comparable with the results obtained from previous experiments, rather than recording the pictures children selected, we recorded children’s preferences based on the pictures they did not select. For example, if a child indicated the “non-integrative picture” as not matching, this was interpreted as a preference for both the “new-set picture” and the “subset picture”. Out of the 22 children who participated in Experiment 4B, the responses of 4 children were excluded due to incorrect answers to the filler items. The analysis of the remaining 18 children’s rejections across the three discourse types (unambiguously new-set, ambiguous, and unambiguously subset) is shown in Figure 9. 

As Figure 9 shows, the same pattern of responses was found in yet another experiment; children strongly preferred the “new-set picture” in ambiguous and unambiguous discourses. The frequency with which children rejected the “new-set picture” was analysed using two 3 (discourse type: unambiguously new-set, ambiguous, and unambiguously subset) × 1 (frequency of rejecting the “new-set picture”) mixed-design ANOVAs. The analysis revealed that there was no statistically significant main effect of discourse type (*F*_1_(2, 34) = 2.22, *p* > 0.05, *η*^2^_p_ = 0.116; *F*_2_(2, 28) = 1.93, *p* > 0.05, *η*^2^_p_ = 0.121), suggesting that children’s likelihood of rejecting the “new-set picture” did not vary significantly across the three types of discourses. As with the results of Experiment 4A, we suggest that the absence of significant effects may be attributed to the relatively small sample size of participants in this experiment, potentially limiting the power to detect differences.

The results of Experiment 4A and Experiment 4B replicated our previous experiments’ findings; children strongly preferred the “new-set picture” across ambiguous and unambiguous contexts. This consistent pattern across experiments rejects the possibility that the effects are artefacts of the task design. While Experiment 4B indicated a tendency among children to reject the “subset picture” less frequently than the “non-integrative picture”, the lack of statistically significant differences across discourse types does not allow for a definitive conclusion. These findings suggest two potential explanations for children’s preferences: first, that children may not recognise that both the “new-set picture” and the “subset picture” accurately represent ambiguous discourses, leading them to favour the “new-set picture”, and second, that children’s difficulty with integrating discourses may predispose them towards a non-anaphoric, non-integrative interpretation corresponding to the “new-set picture”. This observation suggests a potentially nuanced preference in children’s selection behaviour, which requires further exploration.

### 3.5. Experiment 5

#### 3.5.1. Objective

Our experiments reveal a consistent preference among six- to eight-year-old children for the “new-set picture” when interpreting discourses with ambiguous and unambiguous numerically quantified expressions. The results of Experiment 4 suggested that this preference stems from (i) a failure to recognise the possibility of subset readings or (iii) difficulties in integrating the discourse. To further investigate the reasons for this preference, Experiment 5 introduced a picture evaluation task. In this task, children are asked to indicate whether each of the three pictures accurately represents a given discourse. This approach aims to provide insights into the mechanisms driving children’s preference for the “new-set picture”. The key question this addressed was whether children would view only the “new-set picture” as an accurate description of the different discourse types or whether they would also recognise the “subset picture” or perhaps even the “non-integrative picture” as accurate depictions of the described events.

#### 3.5.2. Sample

The participants were 27 children, with a mean age of seven years and three months. 

#### 3.5.3. Materials and Design

In this experiment, children were required to evaluate each of the three pictures, which naturally extended the duration of the experimental procedure. To maintain a similar running time to previous experiments, approximately twenty minutes, it was necessary to reduce the number of discourses. Consequently, we randomly selected and removed one filler item and three experimental discourses, one from each discourse type. Additionally, to mitigate any potential influence of the presentation order on children’s responses, the experimenter randomised the order in which each picture was presented.

#### 3.5.4. Procedure

The procedure was adapted to accommodate the picture evaluation task. Specifically, after the experimenter presented and read the short discourse aloud, children were shown the pictures sequentially, one at a time, for evaluation. Each picture was evaluated individually before proceeding to the next, with all three pictures assessed before moving on to the subsequent discourse. This sequential evaluation ensured a focused assessment of each picture’s relevance to the discourse. Consistent with the approach in Experiment 4, children were reminded that it was possible for more than one picture to accurately represent the discourse, allowing for multiple pictures to be selected as correct descriptions.

#### 3.5.5. Results and Discussion

**Figure 10:**
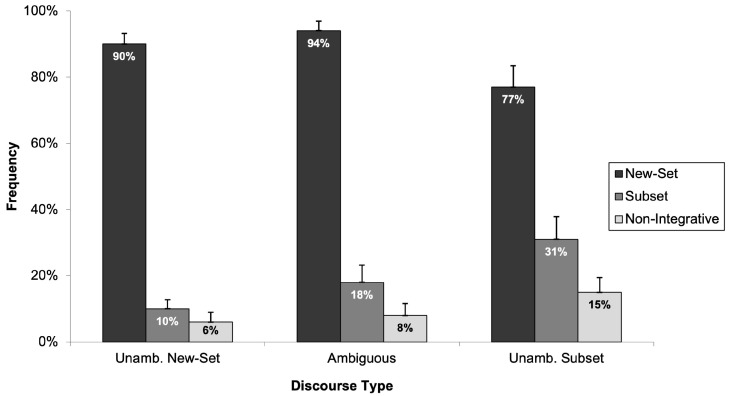
The frequency with which children evaluated each of the three pictures in Experiment 5, with error bars representing the standard error of the mean.

Six of the twenty-seven children were excluded due to incorrect evaluations of the filler items. Thus, analyses were conducted on the responses of 21 children, with a mean age of seven years and three months. Figure 10 shows the frequency with which children evaluated each of the three pictures as accurately reflecting each type. Figure 10 shows a similar pattern of responses to those seen in the previous experiments. Whether interpreting ambiguous or unambiguous numerically quantified noun phrases, children strongly preferred selecting the picture depicting a new-set reading. 

The frequency with which the children selected the “new-set picture” for the three discourse types was put to ANOVA for analysis. Data were analysed using two 3 (discourse type: unambiguously new-set, ambiguous, and unambiguously subset) × 1 (response: evaluation of the “new-set picture” as correctly corresponding to the discourse) mixed-design ANOVAs. The analysis revealed a statistically significant main effect of discourse type (*F*_1_(2, 40) = 6.41, *p* < 0.05, *η*^2^_p_ = 0.243; *F*_2_(2, 22) = 3.95, *p* < 0.05, *η*^2^_p_ = 0.264). Subsequent Tukey HSD post hoc tests were conducted to explore this significance. 

Consistent with our previous experiments, post hoc analysis revealed no significant difference in the rate at which children evaluated the “new-set picture” as matching the discourse between the unambiguously new-set and ambiguous discourse types (*p* > 0.05), suggesting that children perceived the “new-set picture” as equally representative of the discourse in these conditions. Furthermore, there was a statistically significant difference in the frequency of children’s evaluations between the ambiguous and unambiguously subset discourse types (*p* < 0.05), indicating that children were more likely to consider the “new-set picture” as accurate for the ambiguous discourse type compared to the unambiguously subset discourse type. Finally, statistically significant differences were also observed between the two unambiguous discourse types (*p* < 0.05), with children more frequently identifying the “new-set picture” as appropriate for discourses in the unambiguously new-set than in the unambiguously subset discourse type.

The picture evaluation task did not influence children’s response preference for the “subset picture”. Despite the opportunity to evaluate both the “subset picture” and the “new-set picture” as accurately reflecting the discourse, children significantly favoured the “new-set picture” as corresponding to the discourse’s meaning. This finding aligns with our suggestions that children’s difficulty with integration impacts their capacity to comprehend the anaphoric subset reading and that the children’s strong preference for the “new-set picture” does not reflect an interpretation in favour of the new-set reading. 

### 3.6. Experiment 6

#### 3.6.1. Objective

The findings from Experiments 1–5 show a consistent pattern: children aged six to eight years exhibit a strong preference for the “new-set picture” when interpreting ambiguous and unambiguous numerically quantified expressions. The results from Experiments 4 and 5 suggest that a potential underlying factor is difficulty with integration, impeding children’s ability to adopt the subset interpretation. Experiment 6 aimed to further explore this integration challenge by examining children’s responses in the absence of their preferred “new-set picture” option. Specifically, for the discourse types where children’s responses deviated from adult expectations (i.e., ambiguous and unambiguously subset), the “new-set picture” was omitted, forcing children to choose between their less preferred options. In the unambiguously new-set discourse type, the “new-set picture”, which accurately corresponds to the discourse, remained available as an option for children as it matched the discourse’s intended interpretation. Consequently, in this condition, children were given a choice between the “new-set” and “non-integrative” pictures.

This experiment was designed to shed light on children’s interpretive strategies under constrained conditions, focusing on their ability to endorse the subset reading when their preferred option is unavailable. If children predominantly selected the “subset picture” over the “non-integrative picture”, it would suggest they are capable of adopting the subset interpretation under certain conditions. Conversely, a preference for the “non-integrative picture” or no clear choice between these options would highlight the extent of children’s difficulties with integrating discourse elements, indicating that these challenges significantly impede their ability to adopt a subset reading.

#### 3.6.2. Sample

Twenty-nine children, with a mean age of seven years and five months, participated in this experiment.

#### 3.6.3. Materials and Design

We employed a forced-choice picture selection task similar to the one used in Experiments 2 and 4, using the same discourses. Importantly, however, an essential modification was introduced: children were presented with only two pictures and asked to choose between them. In particular, for the ambiguous and unambiguously subset discourse types, children’s preferred “new-set picture” was not included as an option. Instead, children had to choose between the “non-integrative picture” and the “subset picture”. For the unambiguously new-set discourse type, the options given to children were between the “new-set picture” and the “non-integrative picture”. To maintain consistency with the experimental discourses, filler items were adjusted to offer a choice between two pictures. These were the same two pictures used in Experiment 1 (Picture a and Picture b shown in Figure 3), ensuring that children experienced a uniform task structure across both experimental and filler discourses.

#### 3.6.4. Results and Discussion

**Figure 11:**
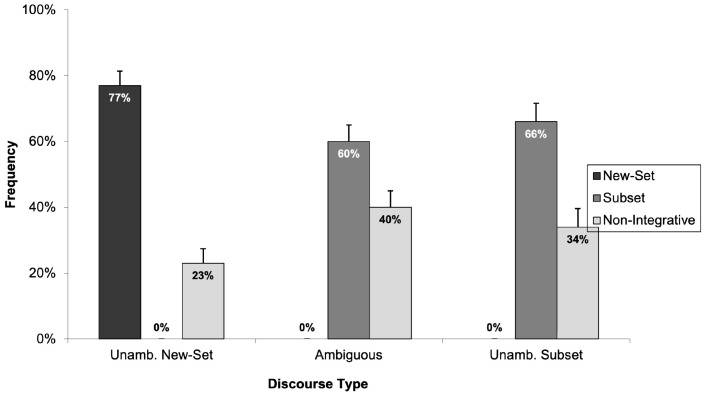
The frequency with which children selected the adult-preferred picture in Experiment 6, with error bars representing the standard error of the mean.

Of the 29 children who participated in this experiment, 4 children failed to correctly evaluate the fillers and/or arithmetic problems, and their responses were excluded from subsequent analysis. The remaining 25 children, with a mean age of seven years and four months, constituted the sample for analysis. To make comparisons across conditions, we analysed how frequently children selected the adult-preferred picture. Specifically, we analysed the frequency with which children selected the “new-set picture” for the unambiguously new-set discourses and the frequency with which children selected the “subset picture” for the ambiguous and unambiguously subset discourses. Data were analysed using two 3 (discourse type: unambiguously new-set, ambiguous, and unambiguously subset) × 1 (response: selection of the “new-set picture” for unambiguously new-set discourse types and selection of the “subset picture” for ambiguous and unambiguously subset discourse types) mixed-design ANOVAs. The analysis revealed a statistically significant main effect of discourse type (*F*_1_(2, 48) = 3.67, *p* < 0.05, *η*^2^_p_ = 0.132; *F*_2_(2, 28) = 4.72, *p* < 0.05, *η*^2^_p_ = 0.252), indicating that children were significantly more likely to choose the “new-set picture” for unambiguously new-set discourses than the “subset picture” for the ambiguous and unambiguously subset discourses. Figure 11 presents the frequency of children’s selections for the adult-preferred picture across the three discourse types. 

Figure 10 clearly illustrates that in the unambiguously new-set discourse type, children’s responses were as expected, with a strong preference for selecting the “new-set picture” as accurately representing the discourse. Interestingly, in the ambiguous and unambiguously subset discourse types, with the “new-set picture” removed, children more frequently selected the “subset picture” over the “non-integrative picture”. To determine whether these selections reflected a genuine preference rather than a random choice, we conducted one-sample *t*-tests comparing children’s selections to a 50% chance level.

The one-sample *t*-test that examined children’s choices in the ambiguous discourse type revealed that children’s selection between the two pictures was not statistically significantly different to the 50% chance level (*t*(24) = 1.99, *p* > 0.05). Thus, without their preferred “new-set picture”, children displayed no clear preference between the remaining options. In the unambiguously subset discourse type, the *t*-test showed a statistically significant preference for the “subset picture” over the 50% chance level (*t*(24) = 2.92, *p* < 0.05), suggesting that when the “new-set picture” is unavailable, children demonstrate a noticeable preference for the “subset picture”. 

The results of Experiment 6 are clear. The findings reveal that children struggle to adopt the subset reading for ambiguous numerically quantified expressions, even when alternative choices are limited. Notably, there was no marked preference for the “subset picture”, which accurately reflected the ambiguous discourse’s intended meaning. Furthermore, the results indicated that when the preferred “new-set picture” was unavailable in unambiguously subset conditions, children were able to adopt the subset interpretation. This suggests that, under certain conditions, children are capable of switching between the two interpretations.

## 4. Discussion

The objective of the present study was to assess whether children, like adults, apply an anaphoric subset interpretation to ambiguous numerically quantified expressions. This is theoretically important because it contributes to a broader understanding of children’s language comprehension. By exploring more complex forms of anaphors, such as numerically quantified expressions, we gain insight into how children’s language processing develops until it starts to resemble that of adults. Through a series of six experiments, our research uncovered a distinct difference in interpretative strategies between adults and children. Contrary to adults, who favour the anaphoric subset reading, six- to eight-year-old children predominantly opted for a non-anaphoric new-set interpretation. This divergence was observed not only for ambiguous numerically quantified expressions but also for discourses that were grammatically disambiguated towards the subset reading (e.g., three cats… two of the cats…). Despite various experimental manipulations—introducing a non-integrative picture option, altering quantifiers to non-numerical, allowing multiple picture selections, and implementing a picture evaluation task—children’s preference for the new-set interpretation persisted. Notably, when the preferred “new-set picture” option was removed, children could assign the anaphoric subset reading in unambiguously subset contexts, yet their decision-making in ambiguous conditions resembled chance, highlighting interpretative challenges. The findings contribute novel insights into children’s comprehension of numerically quantified expressions and their general approach to anaphors, suggesting that children use fundamentally different strategies in discourse comprehension. Moreover, the current study introduces a new systematic approach to examining such phenomena, which can serve as a model for exploring other aspects of language processing.

Before considering the significance of these findings, we must rule out other possible explanations. First, the concern that our observations might be artefacts of the experimental methodology was addressed and rejected. Despite introducing task variations, such as multiple picture selections and a picture evaluation task, children’s strong preference for the new-set picture in both ambiguous and unambiguous contexts persisted, suggesting that children’s preference reflects a genuine interpretive tendency rather than an experimental bias. Second, the notion that children’s selections were based on simple arithmetic strategies was refuted. Even when numerical quantifiers in expressions were replaced with non-numerical quantifiers, children’s preference for the new-set picture remained unchanged, negating the notion that their decisions were based on an arithmetic strategy. Crucially, our findings challenge the conclusions drawn by Wijnen et al. [77], who suggested that young children do not struggle with discourse integration and naturally favour subset readings of ambiguous bare cardinals. Contrary to their assertions, our comprehensive series of experiments consistently demonstrated children’s preference for new-set readings, even in contexts explicitly disambiguated towards subset interpretations. This discrepancy likely stems from methodological differences; notably, our inclusion of control conditions allowed a direct comparison of children’s interpretations under unambiguous scenarios. Thus, it is probable that the subset-reading preference reported by Wijnen et al. reflected a response to their task’s specific demands rather than an inherent interpretive bias, highlighting the importance of experimental design in uncovering the true nature of children’s comprehension strategies.

We propose two possible explanations for the current findings. The first aligns with existing developmental research indicating children’s non-adult-like responses to sentences containing quantifiers, their non-adult interpretations of ambiguous discourses, and their challenges in linking anaphors with the preceding discourse context [11,12,13,14,15,16,17,19,20,21,22,23,24,44,58,59,60,61,62,63,64,65]. This difficulty could reflect a broader issue in discourse comprehension, where children struggle to integrate new information with established contexts. The second explanation relates to a more general cognitive difficulty that young children have in processing set–subset relations, echoing Piagetian observations on how children evaluate relationships between sets of objects [47,48]. Given the data, we cannot distinguish between the two, and thus, both possibilities will be discussed.

Addressing the first possibility for our findings, the difficulty children exhibit in assigning anaphoric interpretations and integrating new information with established discourse contexts stands out. This challenge is not limited to the specific task of interpreting numerically quantified expressions but seems to reflect a broader issue in discourse comprehension among children. Our experiments consistently showed that children favour the picture depicting a new-set reading, even in contexts where the discourse was explicitly disambiguated towards a subset reading. This preference indicates a substantial challenge in recognising that ambiguous discourses can be analysed under both subset and new-set readings. The persistence of children’s preference for the new-set interpretation, even when provided with alternatives that do not align with their favoured choice, suggests a fundamental difficulty in integrating the events described in separate sentences. This challenge is particularly evident in the way children disregard the subset picture, which directly corresponds to an integrated interpretation of the discourse. Such a trend raises questions about the cognitive processes underlying children’s interpretative strategies and whether these processes differ qualitatively from those employed by adults.

Children’s strong preference for the “new-set picture” likely stems from its representation of separate events as non-anaphoric and non-integrative, aligning with their interpretive strategies. This preference highlights a general difficulty in adopting subset readings for numerically quantified expressions, even when grammatical cues point towards such interpretations. Including a non-integrative picture option in our experiments was intended to explore whether children’s selections were influenced by an inclination to interpret the two sentences’ events independently. The results suggest that children’s integration difficulties extend beyond a simple preference for non-integration; they reflect a more profound cognitive challenge in constructing coherent discourse representations that integrate multiple events or pieces of information.

This possible explanation aligns with the existing developmental literature, suggesting that children’s comprehension strategies differ from adults. First, in concordance with studies on children’s use of various anaphors, our experiments demonstrate that children, unlike adults, struggle to employ anaphors effectively to connect succeeding sentences [11,12,13,14,15,16,17,19,20,21,22,23,24]. This difficulty underscores a broader challenge in discourse integration rather than a specific issue with numerically quantified expressions. Second, the results of the current study contribute to the literature on how children comprehend sentences with quantifiers [37,39,42,45,46,52,53,54,55]. We observed an apparent discrepancy between children and adults in interpreting discourses containing numerically quantified expressions, suggesting distinct interpretative strategies across age groups. This divergence emphasises the nuanced ways in which children process quantification in language, contrasting sharply with adult comprehension patterns. Third, our study links to theoretical accounts positing that children do not always represent a set of alternatives in their discourse models [54]. Children’s observed difficulty in integrating events across sentences seems to lead them to prefer a non-integrative, new-set reading over the more complex subset reading. This preference implies a fundamental challenge in conceptualising contrastive sets, particularly evident in their handling of subset interpretations. Lastly, our findings align with research indicating that children incur cognitive costs when revising their initial interpretations of ambiguous sentences [44,45,56,57]. In line with prior observations, our study suggests that children’s difficulty with discourse integration prevents them from reconsidering their initial preference for the new-set reading, even when faced with cues favouring a subset interpretation. This resistance to revision further illustrates the cognitive load associated with integrating and re-evaluating discourse elements among young comprehenders.

Regardless of the detailed account developed above and its many links to other areas that investigate children’s language comprehension, the importance of integrating the effects observed by Piaget and colleagues [47,48] cannot be overlooked. These early findings, which were later replicated by other researchers [49,50,51], provide another explanation for the current findings. Following Piaget’s view, young children often struggle with understanding that a subclass can be part of a broader class, a concept central to our study’s context of numerically quantified expressions. Our research revealed that six- to eight-year-olds consistently preferred the new-set interpretation, a choice that persisted even in contexts unambiguously pointing towards a subset reading. This pattern suggests a fundamental difficulty in processing set–subset relations, akin to the class-inclusion challenges identified by Piaget [47,48]. Notably, when the preferred “new-set picture” was absent, children were more inclined to adopt the subset interpretation in clearly disambiguated scenarios yet remained indecisive in ambiguous conditions. This behaviour aligns with the difficulty in recognising that subclass and class relationships underscore a cognitive challenge beyond mere linguistic interpretation. Therefore, our study extends Piagetian theory by illustrating how difficulties in understanding class–subclass relationships may manifest in children’s discourse processing, emphasising the profound impact of cognitive development on language comprehension.

The current findings do not decisively support one explanation over the other. Furthermore, it remains plausible that children’s strong preference for the “new-set picture” results from a combination of difficulties, both in resolving class–subclass relationships and in integrating the events described across the discourse. Such compounded difficulties could contribute to the observed challenges in children’s assignment of a subset reading to discourses featuring numerically quantified expressions.

The present experiments advance our understanding of children’s interpretative preferences when engaging with discourses that include numerically quantified expressions, offering insights for those interested in children’s language comprehension. While our research provides substantial evidence regarding children’s preferences for new-set interpretations over subset interpretations, it also highlights several limitations that point to avenues for future research. One limitation is our focus primarily on six- to eight-year-old children, leaving unanswered questions about how these interpretative strategies develop before and after this age range. Understanding the developmental trajectory of interpretative biases could offer deeper insights into when and how children’s processing strategies begin to align with those of adults. Furthermore, we did not explore the potential influence of structural principles such as Minimal Lowering and Forward Directionality on children’s interpretations. Future studies could investigate whether and how these principles guide children’s comprehension processes, potentially offering a more nuanced understanding of the cognitive mechanisms at play. Additionally, our experiments were limited in examining the syntactic positioning of quantified noun phrases and bare cardinals. Investigating children’s preferences with varied syntactic presentations could illuminate how sentence structure impacts comprehension and interpretation. Lastly, another potential limitation involves the methodologies employed to assess children’s understanding of expressions that explicitly denote a relationship between two sets, like the partitive construction “of the”. Direct examinations of children’s comprehension in scenarios that more explicitly test their understanding of set relationships could provide clearer evidence of the cognitive processes underlying their interpretative choices. Exploring these areas could not only refine our understanding of children’s processing strategies but also enhance our knowledge of the cognitive and linguistic mechanisms underlying discourse comprehension across development. 

## 5. Conclusions

To conclude, this research represents a comprehensive exploration of how six- to eight-year-old children interpret ambiguous and unambiguous discourses containing numerically quantified expressions. Our findings illuminate the critical role of numerically quantified expressions in textual integration, revealing how these expressions require the integration of information across sentences. Unlike adults, who predominantly adopt an anaphoric subset reading for ambiguous numerically quantified expressions, children strongly prefer a non-anaphoric new-set reading. This preference persists even in contexts explicitly disambiguated towards a subset reading, underscoring a significant developmental divergence in text comprehension strategies. 

These results underscore children’s difficulty linking successive events within a discourse, suggesting a broader challenge in text integration and comprehension. Moreover, our findings resonate with the challenges children encounter in processing class–subclass relationships, further complicating their ability to navigate subset readings effectively. This research contributes to our understanding of the developmental trajectory of language comprehension, highlighting the intricate interplay between cognitive development, linguistic interpretation, and textual integration. It shows that children’s interpretative strategies significantly differ from those of adults, pointing towards a developmental evolution in the processing of numerically quantified expressions and anaphoric references. The significance of this study lies in its contribution to our broader understanding of how children interpret complex anaphors, offering insights into children’s progression towards becoming competent comprehenders.

## Data Availability

Data will be available upon reasonable request from the corresponding author.
